# Enhancement of production of pathogen-suppressing volatiles using amino acids

**DOI:** 10.1016/j.crmicr.2025.100385

**Published:** 2025-04-05

**Authors:** Christina Papazlatani, Annabell Wagner, Zhijun Chen, Hans Zweers, Wietse de Boer, Paolina Garbeva

**Affiliations:** a Netherlands Institute of Ecology (NIOO-KNAW), Department of Microbial Ecology, Droevendaalsesteeg 10, 6708 PB, Wageningen, Netherlands; b Soil Biology Group, Wageningen University & Research, Wageningen, Netherlands; c China Agriculture University, PR China

**Keywords:** Pathogen suppression, Precursors, Amino acids, Volatile organic compounds, *Burkholderia* sp., *Fusarium culmorum*, *Rhizoctonia solani*

## Abstract

Bacterial volatile organic compounds can play a significant role in antagonistic interactions. Enhancing the production of bacterial volatiles that suppress the growth of soil-borne phytopathogenic fungi, has perspective as a sustainable disease control strategy. In the present study, we explored the potential of stimulating *Burkholderia* AD24 and *Paenibacillus* AD87 to produce volatiles that suppress the growth of the plant pathogenic fungi *Fusarium culmorum* PV and *Rhizoctonia solani* AG2.2IIIb. We provided the bacterial strains with a mixture of amino acids that can serve as precursor molecules in metabolic routes leading to emission of suppressive bacterial volatiles. Only *Burkholderia* AD24 was stimulated to produce a volatile blend that led to higher suppression of both pathogens. Subsequent analysis of the volatile composition emitted by *Burkholderia* AD24 in the presence of amino acids, showed higher abundance of antifungal compounds, including sulfur compounds (DMDS), pyrazines (2,5-dimethyl pyrazine) and carbohydrates (3-methyl-1-butanol). Follow-up trials with single amino acids revealed a pathogen specific response effect. When *Burkholderia* AD24 was cultivated in the presence of glutamine and asparagine, the emitted volatile blend suppressed the growth of *F. culmorum*, whereas when cultivated in the presence of glycine, glutamine, arginine and lysine the volatile blend suppressed the growth of *R. solani*. Analysis of the volatile blend composition showed differences between the amino acid treatments. Our findings show that amino acid precursor molecules can stimulate the production of fungistatic volatiles but the sensitivity of the fungal pathogens to these bacterial volatiles varies. This should be considered in future application strategies.

## Highlights

• Amino acids can stimulate the emission of pathogen suppressing volatiles in bacteria.
• Mixed amino acids induced the production of sulfur-, pyrazine-, dioxane- and aromatic compounds in *Burkholderia* AD24.
• Glutamine addition resulted in increased emission of sulfur volatiles.
• Both the identity of the pathogenic fungus and the precursor amino acid impact the effectiveness of volatile suppression.

## Graphical abstract

**Figure:**
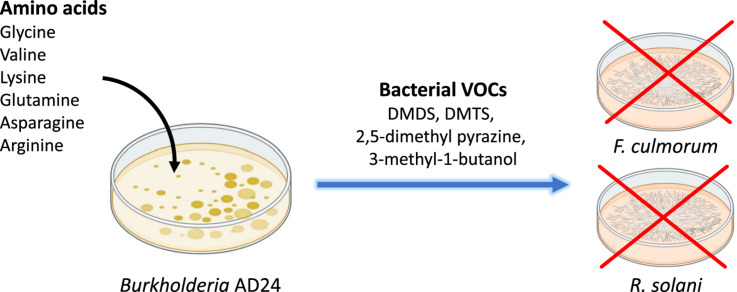

## 1 Introduction

Soil-borne diseases caused by plant pathogenic fungi are responsible for global decrease in yield and quality of crops (Savary et al., 2012), and climate change is exacerbating their deleterious effect (Fones et al., 2020; Singh et al., 2023; Velásquez et al., 2018). These notorious pathogens are commonly treated with synthetic chemical fungicides (Hirooka and Ishii, 2013). However, frequent and indiscriminate use has resulted in the rise of fungicide resistances (Hirooka and Ishii, 2013) and in the development of environmental and public health issues. Therefore, there is need for the development of alternative, sustainable practices to control soil-borne diseases. Various disease management methods have been examined, ranging from agricultural practices to biological control. Agricultural practices for the control of soilborne diseases include the use of (I) different cropping systems, such as mixed cropping, intercropping and crop rotation, to prevent survival and spreading of pathogens, and (II) soil disinfestation methods, like solarization, anaerobic disinfestation, steam sterilization and biofumigation, to eliminate soil-borne pathogenic fungi (Panth et al., 2020). On the other hand, biological control of diseases can involve the application of biocontrol agents (biopesticides), in the form of single strains or microbial consortia, to suppress the soil-borne plant pathogens. The efficacy of these microbes lies in their ability to compete with pathogens for energy sources often including inhibition via release of inhibitors (Chaudhary et al., 2023; Syed Ab Rahman et al., 2018). Nevertheless, the effectiveness of biocontrol agents is still inconsistent as it depends greatly on the establishment and activity of the inoculum under field conditions (Mazzola and Freilich, 2017).

An alternative biocontrol strategy to introducing exogenous strains into the soil, would be to exploit the functional potential of the indigenous soil microbial communities (Chaparro et al., 2012; Mazzola and Freilich, 2017). Inter- and intra- kingdom interactions within soil microbial communities play an important role in community assembly processes (Cornforth and Foster, 2013; Naylor et al., 2022). Competitive interactions between the members of the soil community could result in soil fungistasis, which refers to the prevention of germination of viable fungal propagules or inhibition or retardation of growth of fungal hyphae (Garbeva et al., 2011; Watson and Ford, 2003). Bacterial secondary metabolites play a key role in suppression of soil-borne fungi (Cha et al., 2016), especially volatile organic compounds (de Boer et al., 2019), whose small molecular weight and low polarity allow them to diffuse easily through air- and water-filled pores in the soil and hence participate in chemical interactions between physically separated microorganisms (Tyc Olaf et al., 2017; Weisskopf et al., 2021). Previous studies performed by van Agtmaal et al. have demonstrated the suppressive potential of microbial volatiles in soil against plant pathogenic fungi (van Agtmaal et al., 2018, 2015). Enhancing the production of pathogen suppressing volatiles by indigenous soil bacteria may be a new strategy in biological control of soil-borne diseases.

Soil microbiome stimulating efforts revolve around substrate-mediated recruitment of beneficial microbes or stimulation of pathogen suppressing activities. The vast majority of these studies focus on incorporation in the soil of complex substrates like almond shell compost (Bonilla et al., 2015; Vida et al., 2017, 2016), Brassica seed meal (Cohen et al., 2005; Weerakoon et al., 2012), and chitin amendment (Andreo-Jimenez et al., 2021; Cretoiu et al., 2013). However, the effectiveness of these substrates varies greatly, depending on the type and quality of the organic amendment and the application strategy (Bonanomi et al., 2010, 2007; Clocchiatti et al., 2021; Mayerhofer et al., 2021; Termorshuizen et al., 2006) A more reproducible result could be achieved by adding well-defined materials that induce the desired suppressive activities from microbes. A targeted stimulation of the production of pathogen suppressing volatiles can be achieved via application of precursor molecules of their biosynthetic pathways (Li et al., 2021; Lin et al., 2016; Sun et al., 2009).

As a proof of concept, in this study we aimed to stimulate the production of pathogen suppressing volatiles in the Gram negative *Burkholderia* AD24 and the Gram positive *Paenibacillus* AD87, for it has been shown that their interaction triggered *Paenibacillus* AD87 to produce 2,5-bis(1methylethyl)-pyrazine, which suppressed the growth of plant pathogenic fungi *Fusarium culmorum* PV and *Rhizoctonia solani* AG2.2IIIb (Tyc et al., 2017, 2014). For this purpose, we supplemented the bacteria with a mixture of amino acids that contained precursor molecules involved in the pyrazine biosynthetic pathway (glycine, valine, lysine) and also amino acids that contained a large amount of nitrogen (glutamine, asparagine, arginine) aiming to stimulate the incorporation of the excess nitrogen into pyrazine rings. We hypothesized that the presence of N-rich amino acids would stimulate *Paenibacillus* AD87 to produce higher quantities of the antifungal pyrazine compound. In order to access the production of the pathogen suppressing volatiles, *F. culmorum* and *R. solani* were exposed to the emitted volatile blend. Afterwards, the amino acids were provided separately, in order to determine if the mixture or only a particular amino acid was responsible for the stimulation of suppressive volatile production. Lastly, the volatile blend composition was determined using TD-GC-MS system to identify potential suppressive volatiles produced during amino acid decomposition.

## 2 Materials and methods

### 2.1 Bacterial strain and culture media

The Gram-negative strain, *Burkholderia bryophila* AD24, and the Gram-positive strain, *Paenibacillus xylanexedens* AD87 which were isolated from organic-poor, sandy soil (de Ridder-Duine et al., 2005), were used in this study. The bacterial isolates were routinely cultivated in 0.1 TSBA, (5 gr/L NaCl, 1 gr/L KH_2_PO_4_, 3gr/L Tryptone Soya Broth (TSB), 20 gr/L BACTO agar, pH 6.5) (Garbeva and de Boer, 2009), and, depending on the experimental treatments, 0.1 TSBA was supplemented with 100 ml/L amino acid (AA) mixture stock solution (10x) or 10 ml/L single AA stock solution (100x) after autoclaving. The AA mixture stock solution (10x) concentrations were based on a modified version of the amino acid supplement solution in EZ Rich Defined Medium (EZ Rich Defined Medium - E.coli Genome Project). Briefly, glycine, lysine, valine, glutamine, asparagine and arginine were added in phosphate buffer (pH 6.5) at final concentrations of 8, 4, 6, 6, 4, and 8 mM respectively. The single AA stock solutions (10x) were prepared at 35.7 mM for glycine, lysine and valine, 17.8 mM for glutamine, 20.3 mM for asparagine and 8.9 mM for arginine aiming for 500 mg AA-N per L of 10x stock solution.

### 2.2 Fungal strains and culture media

The plant pathogenic fungal strains *Rhizoctonia solani* AG2.2 III b (Postma and Schilder, 2015) and *Fusarium culmorum* PV (Schmidt et al., 2018) were maintained at the NIOO-KNAW microbial collection. Plant pathogenic oomycete strains *Pythium violae* RC805 and *Pythium sulcatum* RC832 were kindly provided by Bejo company (Warmenhuizen, The Netherlands). All fungal strains were routinely cultivated on 0.5 PDA plates (13.25 gr/L Potato extract - Glucose broth (Carl Roth GmbH), 15 gr/L BACTO agar). For bioassays, the fungal pathogens were first transferred to 0.25 PDA plates, and then to water agar (WA) plates (5 gr/L NaCl, 1 gr/L KH_2_PO_4_, 0.1 gr/L (NH_4_)_2_SO_4_, 20 gr/L agar powder Boom lab, pH 6.5) in order to resemble the nutrient limiting conditions in soil, where they are more susceptible to bacterial volatiles.

### 2.3 Bioassays set up

**Fig. 1:**
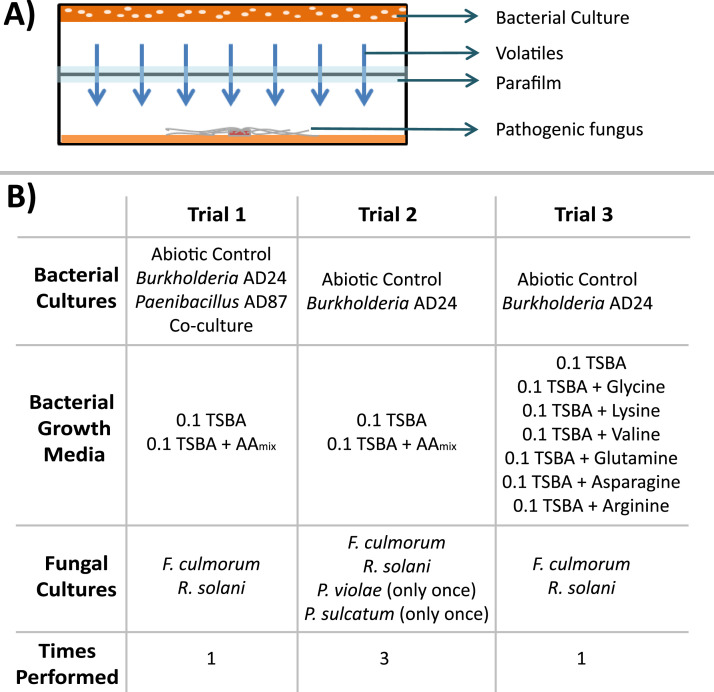
**A)** Schematic representation of the “Bottom-Top” bioassays performed in present study. **B)** Overview of the different trials performed in present study.

Effects of bacterial volatiles on pathogenic fungi/oomycetes were assessed using two Petri-dish bottoms in a so called “bottom-top” approach. This consists of a Petri-dish bottom and an inverted Petri-dish bottom that are connected using parafilm (Fig. 1**A**). This way the bacteria and fungi/oomycetes cultivated in each compartment are not in direct contact but share the same atmosphere. The bottom compartment was used for the growth of plant pathogenic fungi/oomycetes and consisted of WA plates. Firstly, the pathogens were cultivated on 0.25 PDA, at 20 °C, in the dark, for 3 days. Then plugs were cut from the perimeter of the growing mycelium and were used to inoculate WA plates. Transferring the fungal strains from nutrient rich to nutrient poor media was used to increase sensitivity of the pathogens to volatiles. The top compartment was used for bacteria cultivation. Liquid cultures of *Burkholderia* AD24 and *Paenibacillus* AD87 were prepared in 20 ml of TSB and incubated overnight at 24 °C, 160 rpm. The bacterial cultures were adjusted to an optical density OD_600_ of 0.05 and 100μl of the bacterial suspensions were spread in monoculture and co-culture to test plates (0.1 TSBA, 0.1 TSBA + AA_mixture_ or 0.1 TSBA+ AA_single_). The cultures were incubated for 3 days at 20 °C, in the dark. Control plates with no bacteria (abiotic control) were also incubated under the same conditions. Lastly, the 3-day-old bacteria plates were positioned on top of freshly inoculated WA plates and they were secured with two strips of parafilm. The “bottom-top” system was incubated at 20 °C for 3 days before estimating the suppression of *F. culmorum* PV, and 7 days before measuring the suppression of *R. solani* AG2.2 IIIb, *P. violae* and *P. sulcatum*.

Initially, *Burkholderia* AD24 and *Paenibacillus* AD87 were cultivated on 0.1 TSBA and 0.1 TSBA+ AA_mixture_ and *F. culmorum* and *R. solani* were exposed to the emitted volatile blend (Fig. 1**B, Trial 1**). Since, the presence of amino acids stimulated higher suppression of the pathogens by the monoculture of *Burkholderia* AD24 and the co-culture, we proceeded the follow-up experiments only with the Gram-negative bacterium assuming that it was responsible for the increase in suppression in the interaction cultures and because it provided a simpler system to study the stimulation of the production of suppressive volatiles. The effects of volatiles produced by *Burkholderia* AD24 when cultivated on 0.1 TSBA and 0.1 TSBA+ AA_mixture_ were examined 3 times in order to confirm that the presence of amino acids stimulated the production of volatiles that suppressed the growth of both fungal and oomycete pathogens (Fig. 1**B, Trial 2**). After confirmation, growth media supplemented with individual amino acids were prepared in order to pinpoint the amino acid(s) responsible for induction of pathogen suppressing volatiles (Fig. 1**B, Trial 3**).

### 2.4 Determination of pathogen suppression

The growth of *R. solani* AG2.2 III b (except for the first trial; see below) and of the tested oomycetes was estimated by measuring the biomass after 7 days of incubation. The agar containing the pathogen mycelium was transferred to a glass beaker with tap water and melted in a microwave. Then, the fungal biomass was cleaned of any leftover agar by, twice, transferring in a beaker with fresh tap H_2_O and heated in the microwave until boiling. Finally, the clean biomass was transferred into glass tubes with plastic caps with small holes, frozen at -20 °C for at least 16 h and freeze-dried for 24 h. The weight of the empty glass tubes and the glass tubes with dry biomass were recorded.

The growth of *F. culmorum* PV (and for *R. solani* AG2.2 III b only for the first trial) was assessed by measuring the growth area of the mycelium instead of the dry weight because the fungal hyphae were very fragile. After 3 days of incubation in the “bottom-top” system the perimeter of the fungal colony was copied onto transparent sheets, which were then scanned. The fungal growth area was calculated with ImageJ v.1.38 (Schneider et al., 2012).

Suppression of the growth of the tested pathogens when exposed to volatiles produced by *Burkholderia* AD24 was calculated by the following formula:

$$\text{Suppression}=\left(G_{C}-G_{T}\right)/G_{C}$$

where G_C_ corresponds to the average pathogen growth in the controls without exposure to bacterial volatiles and G_T_ corresponds to the pathogen's growth after exposure to bacterial volatiles.

Significant differences in the suppression of tested pathogens that were exposed to the different bacterial volatile blends were estimated by pairwise comparison using Student's *t*-test, if the data were normally distributed, or Wilcoxon rank sum test if not. For multiple treatments, ANOVA accompanied by Tukey's post hoc or Kruskal-Wallis accompanied by Fisher's Least Significant Difference (LSD) test were performed instead. In all cases, Cohen's d effect size (Cohen, 1988) was calculated for each treatment and the corresponding abiotic control. Analysis was performed in R version 4.3.2 (released on 31-10-2023) (R Core Team, 2020).

### 2.5 Determination of effect of the media on *Burkholderia* AD24 growth

After determination of the pathogen suppression on the second and third iteration of Trial 2 and on Trial 3 (Fig. 1), the growth of *Burkholderia* AD24 in the top compartment of each bioassay set up was also determined. The petri dish with the bacterial culture was flooded with 3 ml sterile 0.85 % NaCl solution and the bacterial cells were scraped from the surface of the growth medium with a sterile cell scraper. The bacterial suspension was collected and the surface of the growth medium was further washed with 2 ml sterile 0.85 % NaCl solution, which, upon collection, were combined with the first bacterial suspension. Tenfold dilutions were prepared and 100 μl of dilutions 10^–6^ and 10^–7^ were spread in triplicate on 0.1 TSBA plates for colony counting. The colony forming units (cfu) were determined after 5 days of incubation. Significant differences of the bacterial cfu between 0.1 TSBA and 0.1 TSBA+ AA_mixture_ or 0.1 TSBA+ AA_single_ were determined with Student's *t*-test, if the data were normally distributed, or Wilcoxon rank sum test if not, for the iterations of Trial 2, and ANOVA-Tukey's post hoc or Kruskal-Wallis LSD test for Trial 3 (Fig.1).

### 2.6 Volatile compounds trapping

The volatile blend produced by *Burkholderia* AD24 when cultivated in TSBA and TSBA+AA_mixture_ or TSBA+AA_single_ was examined in order to determine the volatile compounds responsible for suppressive effects. Glass petri dishes with a special lid equipped with an outlet for volatile traps were used (Garbeva et al. 2014). The glass petri dishes were filled with the tested media, inoculated with *Burkholderia* AD24 and incubated for 3 days at 20 °C, in the dark. Control petri dishes with the same media but without bacteria (abiotic controls) were also incubated under the same conditions. Volatile trapping took place in a steel trap containing 200 mg Tenax TA (Camsco, Houton, USA). The traps were placed at the outlet of the glass petri dish lids and volatile compounds were collected via passive diffusion for 24 h, at 20 °C. Afterwards, the traps were gathered, capped and stored at 4 °C until analysis.

### 2.7 GC-MS analysis

The volatiles were measured in a GC-MS system (8890 GC with 5977B MSD, Agilent Technologies Inc, Santa Clara, USA) equipped with an automated thermal desorption unit (TDU; TD100-XR, Markes International LTD, UK) The traps were heated at 280 °C for 8 min so as to release the trapped volatiles, which were then further trapped in a cold trap (-10 ^o^C) in the TDU. Then, 1:10 of the trapped volatiles (split ratio 1:10) were introduced in the GC/MS system by heating the cold trap to 250 °C for 12 min. The temperature program consisted of 1 min at 39 °C and to 315 °C for 10 min^-1^, hold step for 7 min. A constant He flow of 1.2 ml min^-1^ was used. Then the volatile compounds were ionized in electron ionization (EI) mode at 70 eV. Mass spectra were acquired in full-scan mode (33-400 AMU, 4 scans sec^‑1^). An Alkane mix of C8 till C20 (04070-5 ml, Sigma Aldrich) was used to determine the retention index.

### 2.8 Volatilome analysis

The mass spectra of the volatile compounds were visualized with MassHunter Qualitative analysis v. 10.0, build 10305.0 (Agilent Technologies, Santa Clara, USA) and exported as .mzdata xml files for further high throughput data analysis in MZmine v. 3.9 (Schmid et al., 2023). High-throughput analysis was performed on the volatilome of *Burkholderia* AD24 when cultivated on 0.1 TSBA and 0.1 TSBA+ AA_mixture_ or 0.1 TSBA+ AA_single_ and also of the volatiles emitted from the respective growth media (abiotic controls). The GC-MS data were processed according to Automated Data Analysis Pipeline (ADAP) (Jiang et al., 2010) that includes detection of masses, construction of the extracted ion chromatograms (EICs), detection of chromatographic peaks, deconvolution and alignment. Lastly, the final peak (area) intensity table was manually filtered of false compounds (silica peaks, double peaks) and compounds with intensities lower than 1000 or 500 for the dataset of 0.1 TSBA and 0.1 TSBA+ AA_mixture_ or 0.1 TSBA and 0.1 TSBA+ AA_single_ respectively.

### 2.9 Statistical analysis and annotation of volatilome data

Initial normalization of the peak intensity table included pareto-scaling. Partial Least Squares - Discriminant Analysis (PLS-DA; Barker and Rayens 2003) was performed in the normalized peak intensity table to assess the ordination of the volatile samples produced by the abiotic controls and *Burkholderia* AD24 when cultivated in the different media. PLS-DA was carried out with the mixOmics package v. 6.26.0 (Rohart et al., 2017) in R.

Determination of the compounds that were present in significantly higher intensities in *Burkholderia* AD24 volatile blends comparing to the abiotic controls for each growth medium, was achieved via Welch Two Sample *t*-test on square root transformed and pareto-scaled peak intensity table. Compounds with significantly higher intensity (*p*.value < 0.05) in the inoculated treatments, were considered to be produced by *Burkholderia* AD24.

The contribution of the growth medium to the bacterial volatile intensities was removed by subtracting the average raw intensities of each growth medium from the respective bacterial volatile compounds. The “clean” intensities were pareto-scaled and were further subjected to Welch Two Sample *t*-test between 0.1 TSBA and 0.1 TSBA+ AA_mixture_ or 0.1 TSBA+ AA_single_. Statistical analysis code can be found on https://doi.org/10.5281/zenodo.15181543

Annotation of the compounds was performed by matching their mass spectra against the NIST/EPA/NIH EI Mas Spectral library (NIST20) (Naumkin et al., 2023) and the in-house kees library with NIST MS Search program version 2 (NIST Mass Sprectrometry Data Center, National Institute of Standards and Technology, 2020). Final verification of the annotation was performed by comparing the Kovat's retention index of the query and the library compounds.

## 3 Results

### 3.1 Effect of amino acids on production of pathogen suppressing volatiles

**Fig. 2:**
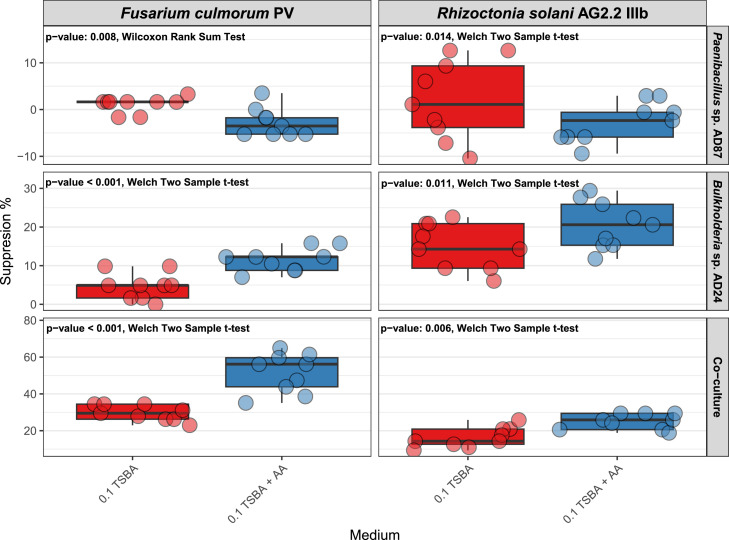
Suppression percentages of *F. culmorum* and *R. solani* after being exposed to volatiles produced by *Paenibacillus* AD87 and *Burkholderia* AD24 when cultivated in monocultures and together in media without (red) or with (blue) the addition of amino acids (AA). Pairwise test and the respective p values are shown in the top right part of each panel (*n* = 9).

Firstly, we tested if the presence of amino acids would be able to stimulate *Burkholderia* AD24 and *Paenibacillus* AD87 monocultures and mixed cultures to produce pathogen suppressing volatiles. *Paenibacillus* AD87 was not capable to suppress the growth of either pathogen regardless of the presence of AA in the growth medium, with suppression levels ranging from 1.09 % ± 1.64 on 0.1 TSBA to −2.73 % ± 3.05 on 0.1 TSBA+ AA_mixture_ for *F. culmorum* and 2.01 % ± 8.57 on 0.1 TSBA and -2.75 % ± 4.3 on 0.1 TSBA+ AA_mixture_ for *R. solani* (Fig. 2). The negative suppression percentage suggests that the growth of the pathogens was stimulated by the volatiles.

On the other hand, for *Burkholderia* AD24 the presence of amino acid mixture in the bacterial growth medium resulted in higher suppression of *F. culmorum*, with an average suppression percentage of 4.74 % ± 3.42 on 0.1 TSBA and 11.5 % ± 3.05 on 0.1 TSBA+ AA_mixture_. Similar results were observed for *R. solani* as well, with average suppression percentages of 15.02 % ± 5.89 and 20.59 % ± 6.18 on 0.1 TSBA and 0.1 TSBA+ AA_mixture_ respectively (Fig. 2).

Likewise, when the two bacteria were cultured together in the presence of amino acids, they exhibited higher suppression levels for both pathogens compared to 0.1 TSBA. Moreover, the suppression levels in 0.1 TSBA+ AA_mixture_ were greater in the co-culture comparing to *Burkholderia* AD24 monoculture in the same medium. In more detail the suppression levels of *F. culmorum* ranged from 29.69 % ± 4.22 on 0.1 TSBA to 51.46 % ± 10.6 on 0.1 TSBA+ AA_mixture_, whereas for *R. solani* the suppression levels raised from 16.3 % ± 5.39 on 0.1 TSBA to 24.90 % ± 4.15 on 0.1 TSBA+ AA_mixture_ (Fig. 2).

Given that the monoculture of *Burkholderia* AD24 was stimulated by the presence of amino acids to produce pathogen suppressing volatiles, we assumed that the Gram-negative bacterium was responsible for the increased suppression in the co-culture, as well. So, we performed the subsequent trials only with *Burkholderia* AD24. In order to confirm that the presence of amino acids promoted the production of suppressive volatiles, the effect of the blend emitted by *Burkholderia* AD24 on 0.1 TSBA and 0.1 TSBA+ AA_mixture_ was examined in 3 more separate iterations (Fig. 1**B, Trial 2**).

**Fig. 3:**
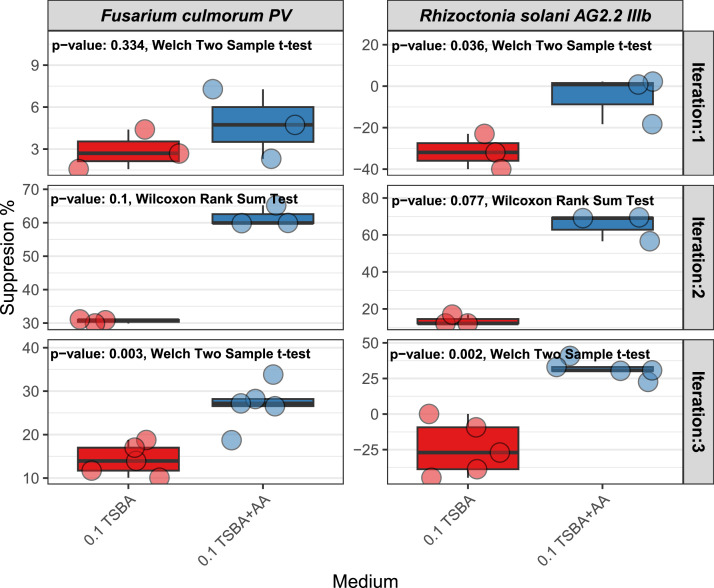
Suppression percentages of *F. culmorum* (right) and *R. solani* (left) after being exposed to volatiles produced by *Burkholderia* AD24 when cultivated on 0.1 TSBA (red) and 0.1 TSBA+ AA_mixture_ (blue), across the different iterations of the 2nd trial. Pairwise test and the respective p values are shown in the top right part of each panel (*n* = 3 for iterations 1 and 2, and *n* = 5 for iteration 3).

*Burkholderia* AD24 was able to suppress the growth of *F. culmorum* on 0.1 TSBA with suppression levels percentages ranging from 2.89 % ± 1.42 to 30.62 % ± 0.64. Nevertheless, the presence of amino acid mixture in the growth medium generally doubled the pathogen's suppression levels which ranged from 4.77 % ± 2.49 to 61.67 % ± 3.06 on 0.1 TSBA+ AA_mixture_ across all trials (Fig. 3). The effect of the amino acid mixture on the suppressive potential of the emitted volatile blend is further supported by an average effect size across trials of -4.9 ± 6.12 (**Supplementary Table 1**).

*R. solani* suppression ranged from -31.64 % ± 8.49 to 15.02 % ± 5.89 when exposed to volatiles produced by *Burkholderia* AD24 on 0.1 TSBA (Fig. 3). In contrast, the presence of amino acids in the bacterial growth medium resulted in suppression percentages ranging from -5.10 % ± 11.47 to 65.07 % ± 7.35 (Fig. 3). Overall, the growth of *R. solani* was hindered by the presence of amino acids in the bacterial medium with an average effect size of -4.17 ± 3.59 in the different trials (**Supplementary Table 1**).

Concerning the oomycetes, the suppression percentages of *Pythium sulcatum* were -23.03 % ± 23.53 and -16.545 % ± 21.52 when exposed to volatiles produced by *Burkholderia* AD24 cultivated on 0.1 TSBA and 0.1 TSBA+ AA_mixture_ respectively (**Supplementary Figure 1**). This indicates that the growth of *P. sulcatum* was, in fact, stimulated by the bacterial volatiles irrespective of the bacterial medium. On the other hand, the growth of *P. violae* was suppressed via volatiles emitted by *Burkholderia* AD24 on 0.1 TSBA with an average suppression of 4.27 % ± 2.65 whereas it was stimulated -1.62 % ± 2.46 on 0.1 TSBA+ AA_mixture_ (**Supplementary Figure 1**).

**Fig. 4:**
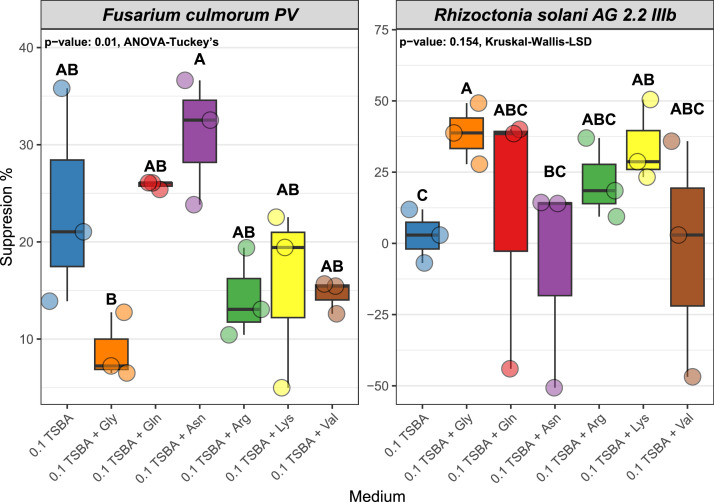
The percentage of growth suppression of *F. culmorum* (right) and *R. solani* (left) when exposed to volatiles produced by *Burkholderia* AD24 that was cultivated on 0.1 TSBA supplemented with various individual amino acids. The statistical test along with the p-value are shown in the top right part of each panel (*n* = 3). Letters indicate significant differences (*p* < 0.05) of the suppression levels between the different media as shown by Tuckey's or Least Significance Difference (LSD) post hoc test.

Addition of single amino acids in 0.1 TSBA had a different effect for each tested pathogen. *F. culmorum* suppression levels on 0.1 TSBA medium were 23.58 % ± 11.18 (Fig. 4). Additions of single amino acids did not result in significantly different suppression values compared to 0.1 TSBA, likely due to high variation of the latter, but the addition of asparagine and glutamine noticeably increased the suppression to an average of 31 % ± 6.54 and 25.85 % ± 0.39 respectively (Fig. 4). The effect size of the difference of suppression levels between 0.1 TSBA and the ones supplemented with asparagine and glutamine were -0.81 and -0.29 respectively, suggesting a large and small effect respectively, according to Cohen (1988) (**Supplementary Table 2**). The presence of the remaining amino acids resulted in suppression percentages of 8.84 % ± 3.42 for glycine, 14.30 % ± 4.61 for arginine, 15.66 % ± 9.36 for lysine and 14.57 % ± 1.71 for valine, which are lower than the suppression levels on 0.1 TSBA control (Fig. 4).

The suppression levels of *R. solani* when exposed to volatiles produced by *Burkholderia* AD24 cultivated on 0.1 TSBA supplemented with glycine and lysine (38.61 % ± 10.72 and 34.13 % ± 14.42 respectively) were statistically significantly higher than 0.1 TSBA without amino acid addition (2.65 % ± 9.39) (Fig. 4). Addition of arginine and glutamine also resulted in suppression levels higher than the control 0.1 TSBA treatment (21.61 % ± 14.06 and 11.51 % ± 48.10 respectively), albeit not statistically significant (p value > 0.05) (Fig. 4). Lastly, no suppression was observed for asparagine and valine (Fig. 4).

### 3.2 Effect of amino acids on *Burkholderia* AD24 growth

Addition of amino acids to 0.1 TSBA growth medium could have an effect in both the production of a different volatile blend and the growth rate of the bacterium. In order to confirm that the higher suppression levels on the media with amino acids (mixture or individual) were due to distinct volatiles and not because of higher numbers of *Burkholderia* AD24, the bacterial cells on the test plates were determined. In most cases, the p value was higher than the threshold of 0.05, which means no differences in bacterial numbers could be determined between 0.1 TSBA and 0.1 TSBA supplemented with AA, either in mixture or individually (**Supplementary Table 3**). The only case where the bacterial numbers were found to be significantly higher in 0.1 TSBA+ AA_mixture_ medium comparing to 0.1 TSBA was in second iteration of Trial 2, on day 6. Spearman correlation test between the bacterial numbers and the suppression levels of second iteration of Trial 2, on day 6 did not show any correlation between the two.

### 3.3 Effect of amino acid mixture on volatile blend composition

High throughput analysis of the volatile blends emitted by *Burkholderia* AD24 and the abiotic controls of the two media resulted in a peak intensity table which, after manual filtering, contained 146 mass features.

**Fig. 5:**
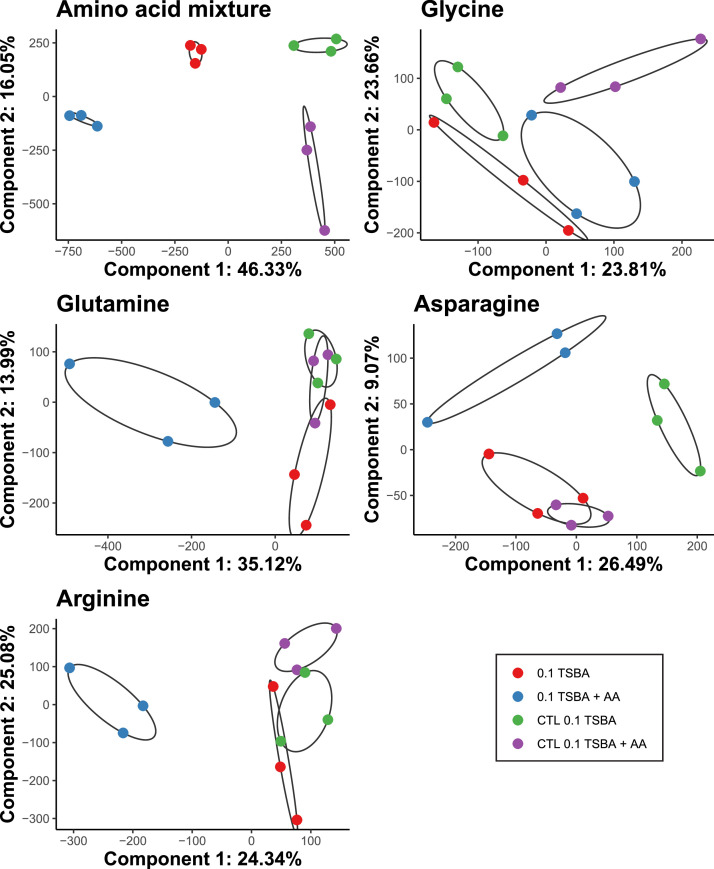
Clustering according to Partial Least Squares - Discriminant analysis (PLS-DA) of the volatile blend emitted by *Burkholderia* AD24 and the abiotic controls (CTL) on 0.1 TSBA (red and green respectively) and 0.1 TSBA+ amino acids (blue and purple respectively).

The volatile blends emitted by *Burkholderia* AD24 when cultivated in 0.1 TSBA and 0.1 TSBA+ AA_mixture_ were clustering separately and apart from the respective abiotic controls (Fig. 5).

Compounds that were detected in significantly higher intensities in the inoculated treatments comparing to the abiotic controls were considered to be produced by *Burkholderia* AD24 (**Supplementary Figure 2**). In total 30 compounds were emitted by *Burkholderia* AD24 irrespective of the growth medium, which consisted of 4 sulfur compounds (dimethyl sulfide @2.09 min, dimethyl disulfide @3.75 min, dimethyl trisulfide @7.20 min, methyl (methylthio)methyl disulfide @9.71 min), a pyrazine compound (2,5-dimethyl pyrazine @6.19 min), 2 heterocyclic oxygen compounds (1-methyl-1,3-dioxolane @ 2.76 min, 1,4-dioxane @3.31 min), 3 aromatic compounds (Benzonitrile @7.42 min, 2-(4‑tert-butylphenoxy)propanoic acid @17.22 min, 2,6-Diphenylphenol @22.48 min), 3 alcohols (2‑methoxy-ethanol @2.64 min, 2-ethoxy ethanol @3.36 min, 3-methyl-1-butanol @3.59 min), 2 alkanes (4-methyl heptane @3.99 min, Alkane Compound @11.50 min),1 alkene (2,4-dimethyl-1-heptene @5.08 min) and 6 compounds that could not be annotated at 4.57 min, 17.54 min, 17.64 min, 17.67 min, 17.73 min and 17.83 min.

These compounds were further subjected into pairwise comparison between the two media that *Burkholderia* AD24 was cultivated, 0.1 TSBA and 0.1 TSBA+ AA_mixture_, in order to verify if their production was stimulated by the presence of amino acids in the bacterial growth medium.

**Fig. 6:**
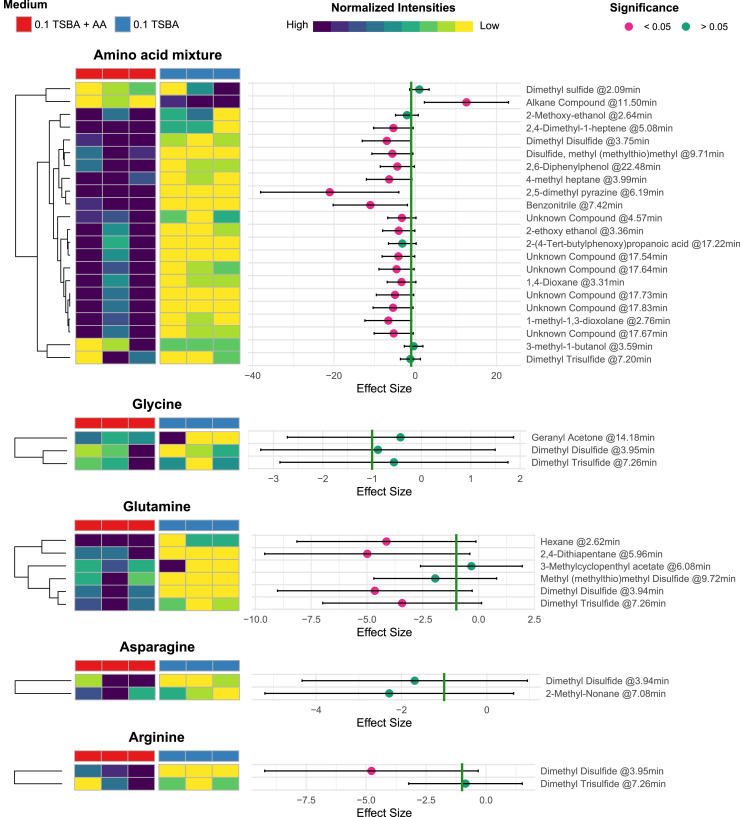
Left: Heatmap of the pareto-scaled intensities ranged between 0 and 1 for each compound that was emitted by *Burkholderia* AD24 when cultivated in 0.1 TSBA+ AA (red, left columns) and 0.1 TSBA (blue, right columns). Middle: Effect size graph of the difference in the intensities of each compound that was produced when *Burkholderia* AD24 was cultivated in the two different media. Points indicate the p value of the Welch Two Sample t-testperformed for each compound (pink: *p* < 0.05, green: *p* > 0.05. Green line represents effect size of −1 standard deviation which is portraying a threshold to strong effect. Left: Annotation and retention time of the compounds emitted by *Burkholderia* AD24.

Overall, the intensities of 2,4-dimethyl heptane, 2,5-dimethyl pyrazine, benzonitrile, 1-methyl-1,3-dioxolane, 1,4-dioxane, 2-ethoxy ethanol, dimethyl disulfide, 4-methyl heptene, methyl (methylthio)methyl disulfide, and of the unknown compounds with retention times 4.57 min, 11.50 min, 17.54 min, 17.64 min, 17.67 min, 17.73 min and 17.83 min were statistically significantly increased by the presence of amino acids (Fig. 6).

Moreover, 2‑methoxy ethanol, dimethyl trisulfide, and 2-(4-Tert-butylphenoxy)propanoic acid exhibited an effect size of less than -1, which signifies strong presence in the 0.1 TSBA+ AA_mixture_, despite the fact that pairwise comparison test was not significant (Fig. 6).

### 3.4 Effect of single amino acids on volatile blend composition

Subsequently, the volatile blend that was produced by *Burkholderia* AD24 when cultivated in the presence of the individual amino acids with the stronger suppressive effect against *F. culmorum* and *R. solani* was also explored. High throughput analysis of the volatile blends emitted by *Burkholderia* AD24 and the abiotic control in 0.1 TSBA and 0.1 TSBA supplemented with glycine, glutamine, asparagine and arginine resulted in peak intensity tables which after manual filtering of false compounds contained 115, 135, 99 and 98 mass features respectively.

The volatile blends emitted by *Burkholderia* AD24 when cultivated in the presence of asparagine, glutamine and arginine were clustered separately, and apart from the blend produced when cultivated on plain 0.1 TSBA and the respective abiotic controls (Fig. 5). On the other hand, the presence of glycine in the growth medium did not result in strong separation between the treatments (Fig. 5).

Pairwise comparison between each inoculated medium and the respective abiotic control showed only a few compounds that were statistically significantly produced by *Burkholderia* AD24, possibly due to high variation of the replicates of each treatment (**Supplementary Figures 3–6**).

Overall, dimethyl disulfide was found to be produced by *Burkholderia* AD24 in all inoculated treatments, irrespective of the growth medium. Dimethyl disulfide @3.95 min, dimethyl trisulfide @7.26 min and geranyl acetone @14.18 min were produced by *Burkholderia* AD24 in slightly higher intensities in the presence of glycine comparing to plain 0.1 TSBA (effect size < 0), but pairwise comparison test did not show any significant differences (Fig. 6). On the other hand, the presence of glutamine significantly stimulated the production of dimethyl disulfide @3.94 min, dimethyl trisulfide @7.26 min, hexane @2.62 min and 2,4-dithiapentane @5.96 min (Fig. 6). Methyl(mehylthio)methyl disulfide @9.72 min and 3-methylcyclopentyl acetate @6.08 min were also found in higher concentrations in the presence of glutamine, albeit not statistically significant (Fig. 6). Asparagine stimulated the emission of 2-methyl nonane @7.08 min and dimethyl disulfide 3.94 min (Fig. 6). Lastly, arginine stimulated the production of dimethyl disulfide @3.95 min and dimethyl trisulfide @7.26 min.

## 4 Discussion

Soil harbors an enormous number and diversity of microorganisms which interact with each other in multiple ways, resulting in either mutualism, commensalism or antagonism. Steering the indigenous soil microbial community towards antagonistic interactions against phytopathogenic fungi, offers a sustainable and eco-friendly alternative to chemical control of soil-borne diseases. In our study we tried to stimulate two model bacteria *Burkholderia* AD24 (Gram negative) and *Paenibacillus* AD87 (Gram positive) to increase the production of volatile compounds that can suppress the growth of the plant pathogenic fungi *Fusarium culmorum* PV and *Rhizoctonia solani* AG2.2 IIIb and the oomycetes *Pythium violae* and *P. sulcatum*.

Tyc et al. (2017) have shown that interspecific interaction between the abovementioned bacteria results in the production by *Paenibacillus* AD87 of the organic volatile compound, 2,5-bis(1methylethyl)-pyrazine, which has broad antimicrobial activity. Contrary to our hypothesis, we found that the provision of pyrazine precursors or amino acids with excess nitrogen, did not enhance the production of the antifungal pyrazine volatile by *Paenibacillus* AD87. On the contrary, cultivation of the bacteria in monocultures and co-culture in growth media supplemented with amino acids showed that only *Burkholderia* AD24 was stimulated to produce suppressing volatiles (Fig. 2). Nevertheless, the suppression levels of both pathogens were significantly higher when exposed to volatiles produced by the co-culture in TSBA+ AA_mixture_ compared to *Burkholderia* AD24 monoculture in the same growth medium. This could be the result of increased suppressive volatile production by both *Burkholderia* AD24 and *Paenibacillus* AD87 when triggered by presence of amino acid mixture and by interaction respectively. Similarly, a stronger suppression of *Fusarium cucumerinum* and *Botrytis cinerea* was found when exposed to the combined volatiles of *Burkholderia vietnamiensis* and *Trichoderma harizanum* co-culture, comparing to the respective mono-cultures (Li et al., 2024). In order to study, in more detail, the effect of amino acid precursors on the release of antifungal volatiles, we have chosen to perform the follow-up experiments only with *Burkholderia* AD24 as it was the bacterium that showed increased suppression with amino acids in the monoculture.

Overall, we observed stronger suppression of both fungal pathogens via bacterial volatiles when *Burkholderia* AD24 was cultivated on TSBA+ AA_mixture_ comparing to plain TSBA. Measuring the bacterial cells on the test plates we showed that the number of bacteria did not change in the presence of amino acids and if it did it was not correlated with the increased suppression levels. The above indicate that the observed enhancement of the suppressive effect was caused by the composition of the volatile blend and not by higher production of similar volatiles due to more abundant bacterial cells.

Consequently, the composition of the volatile blend emitted by *Burkholderia* AD24 in the different media was explored. For this the volatile compounds were collected in stainless steel traps packed with Tenax TA. Tenax TA is a porous polymer resin which has a large range of compound capture (C_6__—_C_30_) (Harshman et al., 2015). Therefore, trapping of volatile compounds with smaller molecular weight is not very effective or reproducible. This is evident from the variation between replicates observed in this study (Fig. 6, Supplementary Figure 2–6).

*Burkholderia* AD24 emitted a wide variety of volatile compounds when cultivated on TSBA including sulfur-, pyrazine-, dioxane- and aromatic compounds and various carbohydrates such as alcohols, and carboxylic acids. A lot of these compounds have already been shown to possess antifungal properties such as dimethyl disulfide, dimethyl trisulfide, acetic acid (Tilocca et al., 2020; Zhao et al., 2022), 3-methyl-1-butanol (Kong et al., 2020) and 2,5 dimethyl pyrazine (Cherniienko et al., 2022). Several of these compounds have also been reported previously in the volatilome of *Burkholderia* species. Acetic acid and dimethyl disulfide were found to be emitted by different *B. tropica* strains when cultivated in PDA (Tenorio-Salgado et al., 2013), while dimethyl disulfide and 2,5-dimethylfuran were reported to be produced by *B. gladioli* BBB-01 (Lin et al., 2021). In our study we observed that the presence of an amino acid mixture in the growth medium enhanced the production of dioxanes (1-methyl-1,3-dioxolane, 1,4-dioxane), sulfur compounds (dimethyl disulfide, dimethyl trisulfide, 2,4-dithiapentane, methyl (methylthio)methyl disulfide, pyrazines (2,5-dimethyl pyrazine), aromatic compounds (Benzonitrile), 2-(4-Tert-butylphenoxy) propanoic acid and 2,4-dimethyl-1-heptene (Fig. 4). The mixture consisted of amino acids that could stimulate the production of pyrazine volatile compounds. Even though, increased production of 2,5-dimethyl pyrazine has been shown in this study, it is also evident that the presence of amino acids stimulated the whole bacterial metabolism which resulted in increased emission of volatiles from various chemical groups.

Consequently, we applied each amino acid separately in order to pinpoint which one was responsible for the increase in emission of pathogen suppressing volatiles. Supplementing 0.1 TSBA with asparagine resulted in increased production of 2-methyl nonane and dimethyl disulfide, whereas arginine stimulated the production of dimethyl disulfide and dimethyl trisulfide. Interestingly, addition of glutamine in the growth medium resulted in increase of the production of several sulfur compounds such as dimethyl disulfide, dimethyl trisulfide, 2,5-dithiapentane and methyl(methylthio)methyl disulfide. Similar to our findings, Zhu et al. (2023), in their study of the effect of glutamine addition on sensory properties of Chardonnay wine, also found significant increase in dimethyl disulfide comparing to the untreated control. The first step of glutamine metabolism towards glutamate is performed by glutamine transaminase (GT), which transforms l-glutamine into 2-oxoglutaramate by transferring the amino group into a pyruvate molecule. There is evidence that GT could also participate in the last step of the methionine salvage pathway in mammals, plants and bacteria, in which it transfers the amino group from l-glutamine to 4-Methylthio-2-oxobutanoic acid instead of pyruvate, therefore producing methionine (Ellens et al., 2015), which in turn is a precursor amino acid to numerous sulfur volatile compounds (Landaud et al., 2008; Sreekumar et al., 2009; Yu et al., 2022). Moreover, glutamine provision could have a stimulating effect on the overall bacterial metabolism as it acts as a nitrogen donor in various biosynthetic reactions, including amino acids metabolism (Dai et al., 2013) and various transamidation reactions (Griffin et al., 2002), or it acts as a signaling molecule in bacterial interactions (Forchhammer 2007).

The volatile blend emitted by *Burkholderia* AD24 when cultivated on TSBA supplemented with amino acids was examined in a set up where the bacterium was not exposed to the volatiles emitted by the fungal pathogens. Fungi emit chemically diverse volatiles (Razo-Belmán et a., 2023) that act as info-chemicals, contributing significantly to interactions with other microorganisms or plants (Duc et al., 2022). Several studies have shown that fungal volatiles affect the bacterial responses (Spraker et al., 2014; Schmidt et al., 2016, 2017; Jones et al., 2017). In present research, while the bioassay set up allowed for diffusion of fungal volatiles that could potentially influence the bacterial response, the volatile trapping set up included only *Burkholderia* AD24 as the aim of the study was to focus on the use of precursor molecules to stimulate bacterial production of antifungal volatiles.

The suppressive potential of the volatile blend emitted by *Burkholderia* AD24 was not only influenced by the presence of additional amino acids in the bacterial growth medium, but also by the identity of the pathogen that was exposed to it. In our assays, *R. solani* was affected the strongest by the bacterial volatile blend produced in the presence of the amino acid mixture, followed by *F. culmorum*, whereas no suppression whatsoever was observed for the tested oomycete strains (Fig. 3). Similar to our results, several studies have reported a pathogen-specific suppressive effect of volatile blends emitted by bacteria (Garbeva et al., 2014; de Boer et al., 2019) and specifically members of the *Burkholderia* genus (Groenhagen et al., 2013; Tenorio-Salgado et al., 2013; Chen et al., 2020).

Present study focused on steering isolated soil bacteria to enhance production of volatile compounds that can suppress plant pathogenic fungi, ultimately aiming to develop a strategy for sustainable disease control. Application of precursor molecules can be achieved via seed coating. Amirkhani et al., developed a method to deliver bio-based biostimulants, like vermicompost and soy flour, as seed coatings (Amirkhani et al., 2019). Nevertheless, it should be noted that application of precursor molecules in soil, could have positive or detrimental effects to the composition and functions of non-target microbial communities, highlighting the need for further studies under realistic application schemes.

## 5 Conclusions

In present work we showcased the potential of steering soil bacteria to produce volatiles with antifungal properties by providing them with precursor molecules in the form of amino acids. The volatile blend produced by *Burkholderia* AD24 supplemented with amino acids, revealed the increase in production of sulfur, pyrazine, dioxane and aromatic compounds most of which possess antifungal properties. Moreover, application of glutamine alone in the bacterial growth medium resulted in increase in the emission of sulfur volatiles, possibly via stimulation of biosynthetic pathways that involve the production of methionine. Our results demonstrate that bacterial interspecific interactions, the fungal pathogen susceptibility to the emitted volatiles and the choice of precursor molecules, can have an impact on the effectiveness of stimulating bacteria to produce suppressive volatiles, and they should be taken into consideration in future application scenarios.

## Funding sources

This work was supported by the Dutch Research council (NWO) through the Open Technology Programme ‘Connecting Innovators’ (18728 TTW VOLControl)

## CRediT authorship contribution statement

**Christina Papazlatani:** Conceptualization, Data curation, Formal analysis, Visualization, Investigation, Writing – original draft. **Annabell Wagner:** Methodology, Investigation. **Zhijun Chen:** Investigation. **Hans Zweers:** Methodology, Investigation. **Wietse de Boer:** Conceptualization, Funding acquisition, Writing – review & editing. **Paolina Garbeva:** Conceptualization, Funding acquisition, Project administration, Writing – review & editing, Supervision.

## Declaration of competing interest

The authors declare that they have no known competing financial interests or personal relationships that could have appeared to influence the work reported in this paper.

## Data Availability

Data will be made available on request.

## Appendix Supplementary materials

Image, application 1
