# Supplementary material for: Enhancement of production of pathogen-suppressing volatiles using amino acids
Source: Curr Res Microb Sci. 2025 Apr 5;8:100385. doi: 10.1016/j.crmicr.2025.100385 (PMC12018582; doi:10.1016/j.crmicr.2025.100385)
Supplement: Supplementary file 1 [file mmc1.pdf]

Supplementary Table 1. Cohen's d effect size with lower and upper confidence intervals, and pairwise test with the corresponding p-value of the suppression levels of *F. culmorum* PV and *R. solani* AG2.2 IIIb between 0.1 TSBA and 0.1 TSBA + AA across the different iterations of Trial 2.

Experiment	Pathogen	eff.size	CI.lower	CI.upper	stat.test	pvalue
Iteration:1	Fusarium culmorum PV	-0.93	-3.31	1.46	Welch Two Sample t-test	0.334
	Rhizoctonia solani AG2.2 IIIb	-2.63	-5.72	0.47	Welch Two Sample t-test	0.036
Iteration:2	Fusarium culmorum PV	-14.06	-25.56	-2.57	Wilcoxon rank sum exact test	0.100
	Rhizoctonia solani AG2.2 IIIb	-9.24	-16.99	-1.50	Wilcoxon rank sum test with continuity correction	0.077
Iteration:3	Fusarium culmorum PV	-2.74	-4.77	-0.71	Welch Two Sample t-test	0.003
	Rhizoctonia solani AG2.2 IIIb	-3.89	-6.38	-1.41	Welch Two Sample t-test	0.002
	Pythium sulcatum RC832	-0.29	-1.75	1.18	Welch Two Sample t-test	0.661
	Pythium violae RC805	2.30	0.42	4.18	Welch Two Sample t-test	0.007

Supplementary Table 2. Cohen's d effect size with lower and upper confidence intervals of the suppression levels of *F. culmorum* PV and *R. solani* AG2.2 IIIb between 0.1 TSBA and 0.1 TSBA supplemented with each AA individually

Pathogen	Tested Medium	Effect size	C.I. Lower	C.I. Upper
Fusarium culmorum PV	0.1 TSBA + Arg	1.09	-1.34	3.51
	0.1 TSBA + Asn	-0.81	-3.17	1.55
	0.1 TSBA + Gln	-0.29	-2.57	1.99
	0.1 TSBA + Gly	1.78	-0.90	4.46
	0.1 TSBA + Lys	0.77	-1.58	3.12
	0.1 TSBA + Val	1.13	-1.31	3.57
Rhizoctonia solani AG 2.2 IIIb	0.1 TSBA + Arg	-1.59	-4.19	1.01
	0.1 TSBA + Asn	0.37	-1.92	2.66
	0.1 TSBA + Gln	-0.26	-2.53	2.02
	0.1 TSBA + Gly	-3.57	-7.22	0.08
	0.1 TSBA + Lys	-2.59	-5.66	0.48
	0.1 TSBA + Val	0.18	-2.09	2.45

Supplementary Table 3 The mean cfu ± standard deviation (SD) of *Burkholderia* AD24 at the end of the 2nd and 3rd iteration of Trial 2 and Trial 3. The pathogen of the bioassay as well the total incubation time of the bacterium is shown. The effect size with lower and upper confidence intervals, and statistical test with the corresponding p-value of the cfus of *Burkholderia* AD24 between 0.1 TSBA and 0.1 TSBA + $AA_{mixture}$, or between 0.1 TSBA and 0.1 TSBA + each single amino acid are also included

Experiment	Bioassay	Days after inoculation	Growth Medium	Mean cfu ± SD	Effect size	CI.lower	CI.upper	Statistical test	pvalue
Trial 2: Iteration 2	Fusarium culmorum PV	Day 6	0.1 TSBA	5.0E+07 ± 5.0E+07	-3.61	-7.29	0.06	Welch Two Sample t-test	0.036
			0.1 TSBA + AAmix	5.17E+08 ±1.76E+08					
	Rhizoctonia solani AG2.2 IIIb	Day 10	0.1 TSBA	1.60E+09 ± 4.78E+08	2.04	-0.76	4.83	Welch Two Sample t-test	0.112
			0.1 TSBA + AAmix	8.81E+08 ± 1.40E+08					
Trial 2: Iteration 3	Fusarium culmorum PV	Day 6	0.1 TSBA	1.27E+09 ±8.87E+07	1.15	-0.42	2.73	Welch Two Sample t-test	0.123
			0.1 TSBA + AAmix	1.09E+09 ± 2.04E+08					
	Rhizoctonia solani AG2.2 IIIb	Day 10	0.1 TSBA	5.88E+08 ± 7.96E+07	3.02	0.89	5.15	Welch Two Sample t-test	0.001
			0.1 TSBA + AAmix	3.55E+08 ± 7.49E+07					
	Pythium sulcatum RC832	Day 10	0.1 TSBA	8.17E+08 ± 1.63E+08	0.29	-1.18	1.75	Welch Two Sample t-test	0.661
			0.1 TSBA + AAmix	7.65E+08 ± 1.96E+08					
	Pythium violae RC805	Day 10	0.1 TSBA	1.18E+09 ± 1.97E+08	0.05	-1.41	1.51	Welch Two Sample t-test	0.938
			0.1 TSBA + AAmix	1.16E+09 ± 4.13E+08					
Trial 3	Fusarium culmorum PV	Day 6	0.1 TSBA	1.05E+09 ± 2.85E+08	0.00	0.00	1.00	ANOVA Tukey	0.572
			0.1 TSBA+Glycine	1.24E+09 ± 4.95E+08					
			0.1 TSBA+Glutamine	1.14E+09 ± 2.85E+08					
			0.1 TSBA+Asparagine	1.03E+09 ± 2.60E+08					
			0.1 TSBA+Arginine	1.11E+09 ± 2.30E+08					
			0.1 TSBA+Lysine	1.05E+09 ± 3.53E+08					
			0.1 TSBA+Valine	7.12E+08 ± 1.75E+08					
	Rhizoctonia solani AG2.2 IIIb	Day 10	0.1 TSBA	9.23E+08 ± 1.42E+08	0.10	0.00	1.00	ANOVA Tukey	0.292
			0.1 TSBA+Glycine	1.09E+09 ± 1.16E+08					
			0.1 TSBA+Glutamine	1.19E+09 ± 1.77E+08					
			0.1 TSBA+Asparagine	1.07E+09 ± 1.85E+08					
			0.1 TSBA+Arginine	1.24E+09 ± 1.14E+08					
			0.1 TSBA+Lysine	1.20E+09 ± 2.58E+08					
			0.1 TSBA+Valine	1.23E+09 ± 1.38E+08					

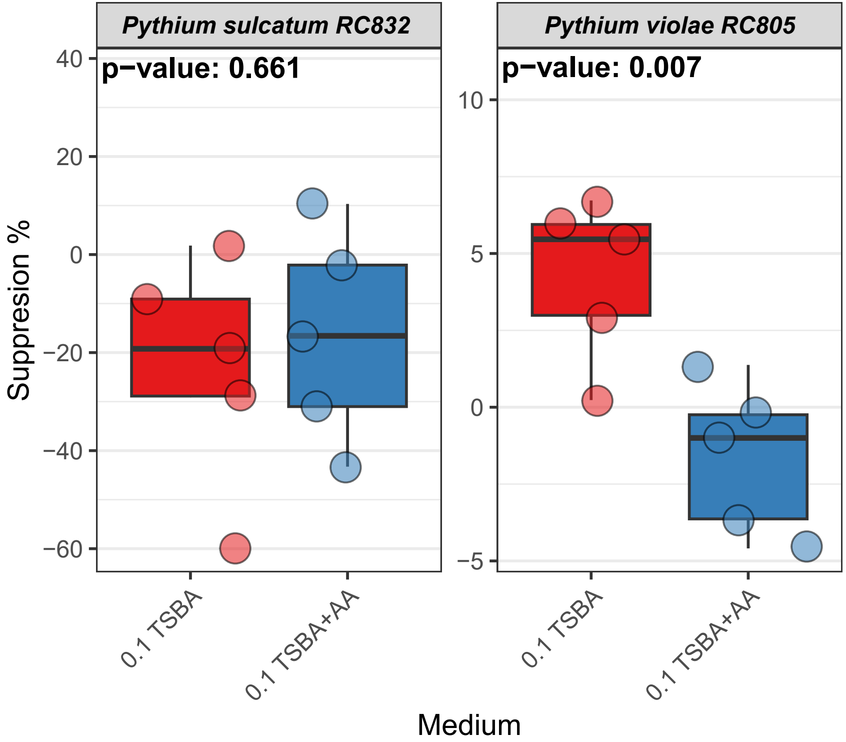

Supplementary Figure 1. Suppression percentages of *P. sulcatum* and *P. violae* after being exposed to volatiles produced by *Burkholderia* AD24 when cultivated on 0.1 TSBA (red) and 0.1 TSBA + $AA_{mixture}$ (blue)

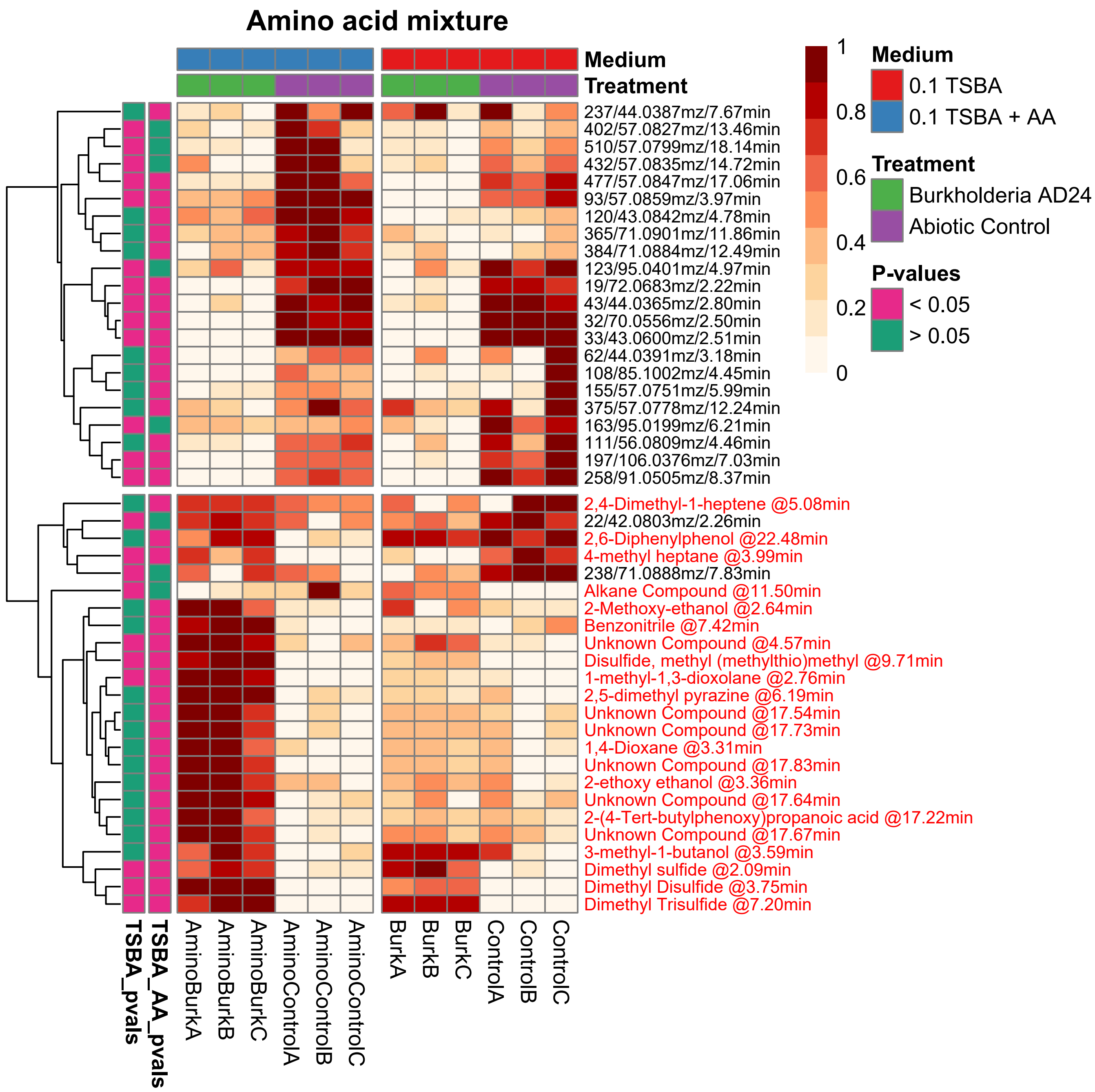

Supplementary Figure 2. Heatmap of transformed intensities (square root transformed, pareto scaled, ranged between 0 and 1) of the compounds that were found to be significantly different between inoculated treatments and abiotic controls. Compound names in red indicate that they were found in significantly higher intensities in inoculated media. Left most columns indicate p-value of the pairwise comparisons for each compound in 0.1 TSBA (right) and 0.1 TSBA + AAmixture (left).

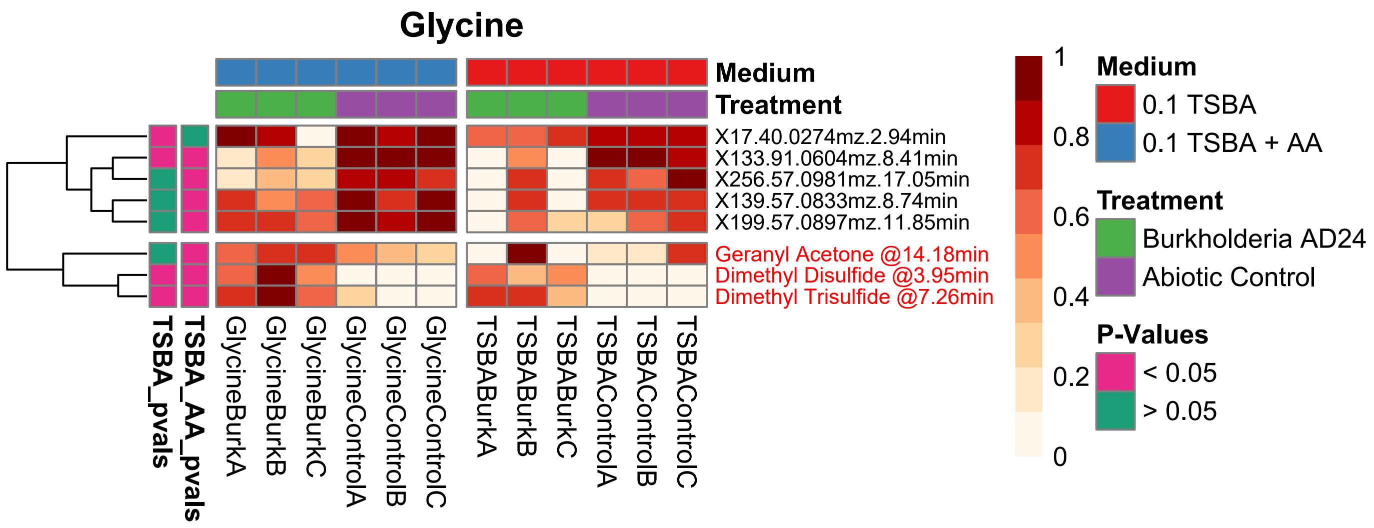

Supplementary Figure 3. Heatmap of transformed intensities (square root transformed, pareto scaled, ranged between 0 and 1) of the compounds that were found to be significantly different between inoculated treatments and abiotic controls. Compound names in red indicate that they were found in significantly higher intensities in inoculated media. Left most columns indicate p-value of the pairwise comparisons for each compound in 0.1 TSBA (right) and 0.1 TSBA + glycine (left).

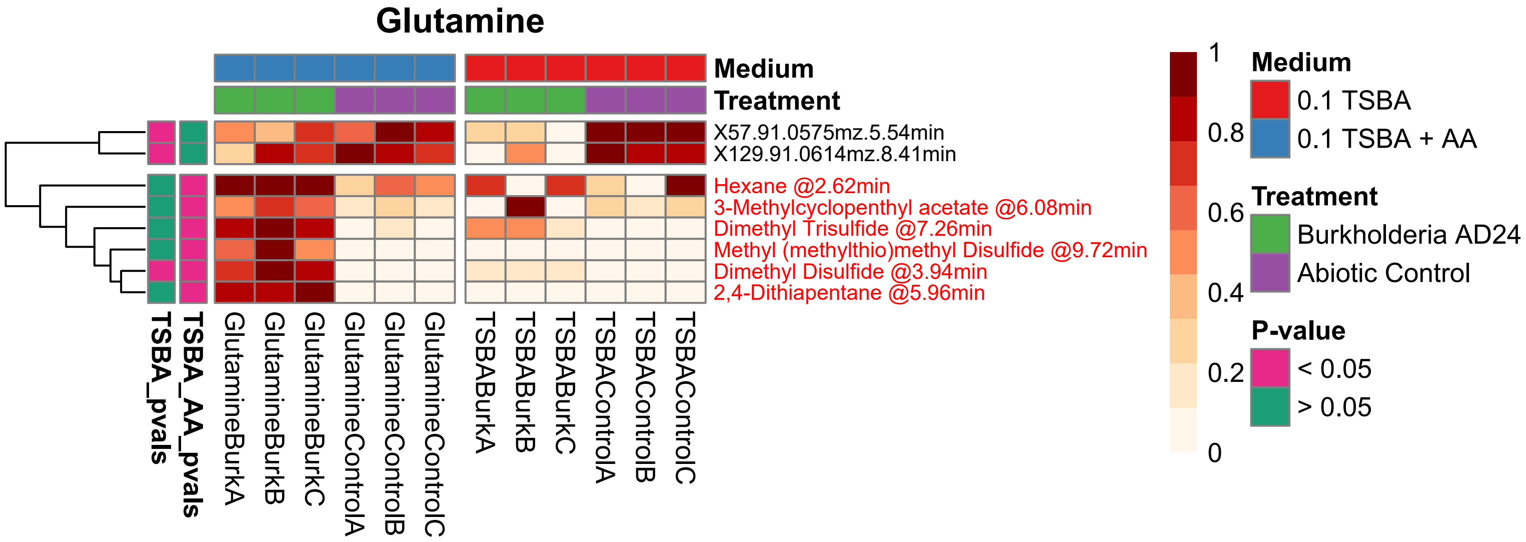

Supplementary Figure 4. Heatmap of transformed intensities (square root transformed, pareto scaled, ranged between 0 and 1) of the compounds that were found to be significantly different between inoculated treatments and abiotic controls. Compound names in red indicate that they were found in significantly higher intensities in inoculated media. Left most columns indicate p-value of the pairwise comparisons for each compound in 0.1 TSBA (right) and 0.1 TSBA + glutamine (left).

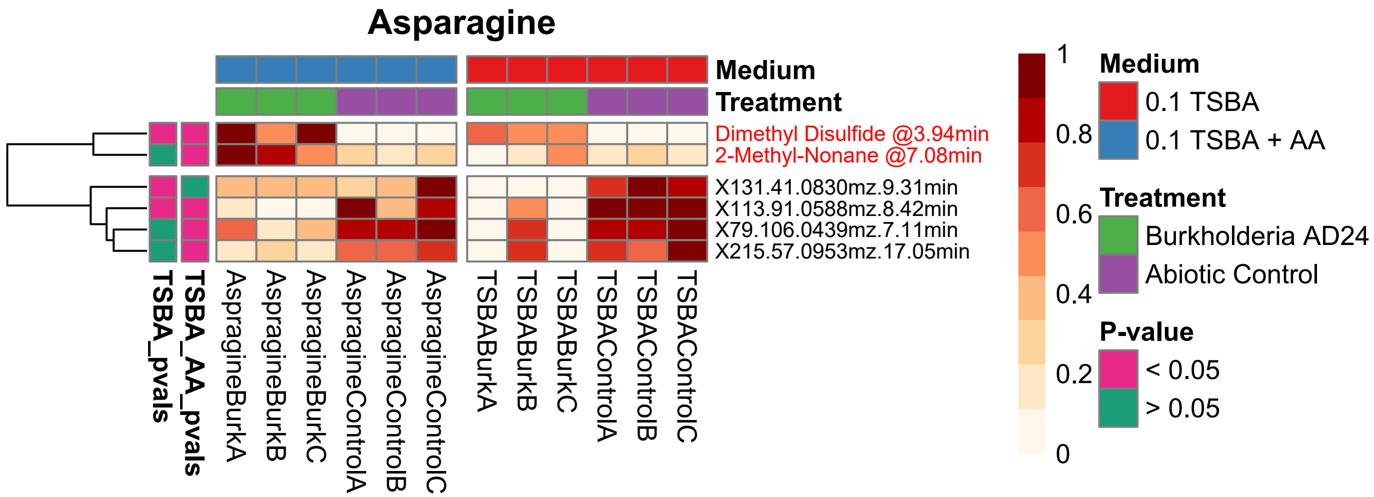

Supplementary Figure 5. Heatmap of transformed intensities (square root transformed, pareto scaled, ranged between 0 and 1) of the compounds that were found to be significantly different between inoculated treatments and abiotic controls. Compound names in red indicate that they were found in significantly higher intensities in inoculated media. Left most columns indicate p-value of the pairwise comparisons for each compound in 0.1 TSBA (right) and 0.1 TSBA + asparagine (left).

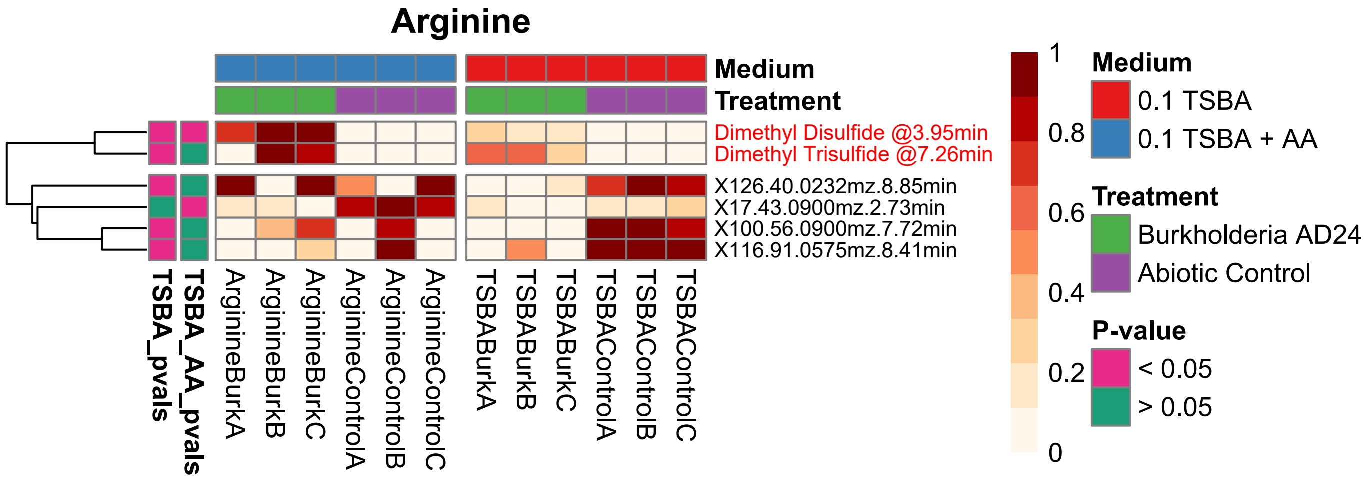

Supplementary Figure 6. Heatmap of transformed intensities (square root transformed, pareto scaled, ranged between 0 and 1) of the compounds that were found to be significantly different between inoculated treatments and abiotic controls. Compound names in red indicate that they were found in significantly higher intensities in inoculated media. Left most columns indicate p-value of the pairwise comparisons for each compound in 0.1 TSBA (right) and 0.1 TSBA + arginine (left).
